# Clinical profile of adult stroke patients in Angola: a cross-sectional study

**DOI:** 10.1038/s41598-025-88288-7

**Published:** 2025-03-28

**Authors:** Herculana Artur Gonçalves, João Adilson Gama Ricardo

**Affiliations:** 1 Physiology Department, Faculty of Medicine, Agostinho Neto University, Luanda, Angola; 2 Neurology Service, Clínica Sagrada Esperança, Luanda, Angola

**Keywords:** Stroke, Profile, Risk factors, Angola, Diseases of the nervous system, Neuro-vascular interactions, Neuroscience, Neurology, Cerebrovascular disorders, Stroke

## Abstract

Stroke is a leading cause of death and disability worldwide, and the greatest burden of this disease has been observed in low- and middle-income countries, which continue to face several challenges in stroke care. The objective of this study was to identify the clinical profile of Angolan stroke patients in a tertiary hospital in Luanda. A cross-sectional investigation was conducted at Clínica Sagrada Esperança in Angola a tertiary center, on stroke patients admitted to the neurology service. Data was collected from November 2022 to March 2023. We included all patients who were admitted to the emergency department. Patients under 18 years of age, who had a previous stroke ≥ 2 score on the modified Rankin scale (mRS), and had brain tumors were excluded. We identified 139 stroke patients. The mean age was 59.5 ± 13.5 years, most were male (65.5%). The ischemic stroke was the most prevalent subtype (73.4%). The mean length of stay was 8.3 ± 4.7 days. Higher mRS scores at discharge were associated with complications (*p* < 0.001) and prolonged hospitalization (*p* = 0.001). The most frequent risk factors were hypertension (96.5%), alcohol use (67.4%), and diabetes (29.6%). Our study provides crucial insights into the profile of stroke patients in Angola. The collected data are vital for informing effective preventive measures and improving patient care.

## Introduction

Stroke is one of the world’s leading causes of death, incapacity, and dementia^1^.

The definition and management have been improving with scientific developments. It used to be based on sudden and focal neurological symptoms in the context of an important ischemia or hemorrhagic process occurring in the brain, spinal cord, or even retina^2^. Nowadays, stroke is a pathological problem with a wide range of clinical features, in the background of a block of the brain blood flow that leads to brain neurons death, associated with alterations on the imaging exam^3,4^.

It is estimated that approximately 80% (80%) of all stroke events are caused by the ischemic type, and the remaining 20% (20%) of strokes are caused by hemorrhagic strokes, of which only 5% (5%) are subarachnoid hemorrhages^5^.

Although its incidence has declined, it continues to rank as the fifth most significant cause of death in the United States of America, a high-income country with immense socioeconomic influence^6^. Africa has experienced a consistent rise in the incidence of this disease, with an estimated 316 cases per 100,000 people, which is among the highest rates observed worldwide^1^.

African countries are currently experiencing an epidemiological transition as a result of substantial transformations in socioeconomic and lifestyle determinants. The prevalence of noncommunicable chronic diseases has increased because of this transition^7^. Therefore, it is critical to evaluate the current epidemiological and clinical parameters in Africa, considering the continent’s distinctive regional and population-specific characteristics that distinguish it from other continents.

Despite substantial scientific advancements and increased understanding of stroke, it continues to be a pathological condition with a very negative impact, especially among low- and middle-income countries^8^.

At present, it constitutes a worldwide public health concern that has substantial socioeconomic repercussions for both national healthcare systems and patients^9^.

An understanding of stroke risk factors enables healthcare services to adopt a preventive stance, thereby decreasing the prevalence of stroke.

The lack of previous research and results on this topic highlights the importance of this study and its potential to provide valuable insights into stroke management, especially in Angola.

Our study aimed to examine the sociodemographic and clinical profiles of Angolan stroke patients at a tertiary hospital.

## Methods

We performed a cross-sectional study on patients with stroke admitted to the neurology service at Clínica Sagrada Esperança (CSE), located in Luanda, Angola, from October 2018 to March 2022.

CSE is a tertiary hospital, and one of the largest healthcare institutions in the country and provides equitable care to patients of all social statuses. It provides a wide range of general care services, including several medical specialties, as well as 24-hour emergency medical services and complete laboratory facilities. It boasts a capacity of over 329 beds, with the Neurology service being a part of the Medicine Department.

We included all patients that attended the emergency department diagnosed with stroke confirmed by computed tomography (CT) and/or magnetic resonance imaging (MRI). The definition of stroke used for diagnosis was based on the criteria established by the American Heart Association (AHA) and American Stroke Association (ASA)^10^, an episode of neurological disfunction caused by focal cerebral, spinal, or retinal infarction (ischemic) or a focal collection of blood within the brain parenchyma, ventricular system or subarachnoid space (hemorrhagic), which in not due to trauma and is confirmed by imaging. Patients who were under 18 years old, had a previous stroke score ≥ 2 points on the modified Rankin scale (mRS), cerebral venous sinus thrombosis and had brain tumors were excluded.

All patients underwent Doppler ultrasonography of the carotid arteries and blood tests, including a coagulation panel, complete blood count (CBC), and biochemical assessments for renal and liver function. The majority also had transthoracic echocardiography (TTE), while a few of them received CT angiography or MR angiography.

### Variables and data collection

The main variables studied were age, sex, occupation, risk factors, stroke subtype, symptoms, blood pressure at admission, symptom onset time at hospital arrival, mRS score at discharge moment, length of stay, and clinical complications during hospital stay.

The data were collected from the Neurology Service database and the hospital’s software to identify patients who were admitted through the emergency department with the International Classification of Diseases (ICD). Data collection was conducted between November 2022 and March 2023.

### Statistical analysis

The collected data were subsequently entered into a database that was subsequently analyzed utilizing the IBM Statistical Package for the Social Science (SPSS) for Windows software version 25.0 (Armonk, NY: IBM Corp; 2017).

Qualitative variables were analyzed with the Chi-square test (X^2^), and group comparisons were performed using the Mann-Whitney U test, with statistical significance defined as *p* < 0.05.

### Ethical approval

The protocol of this study was approved by the ethical and scientific committees of both, the Faculty of Medicine of Agostinho Neto University and Clínica Sagrada Esperança. We followed the ethical guidelines outlined in the Helsinki Declaration for conducting research involving human subjects. We guaranteed the protection of data anonymity and confidentiality and utilizing it solely for purposes stated above. Informed consent was obtained from all subjects and/or their legal guardian(s).

## Results

**Fig. 1:**
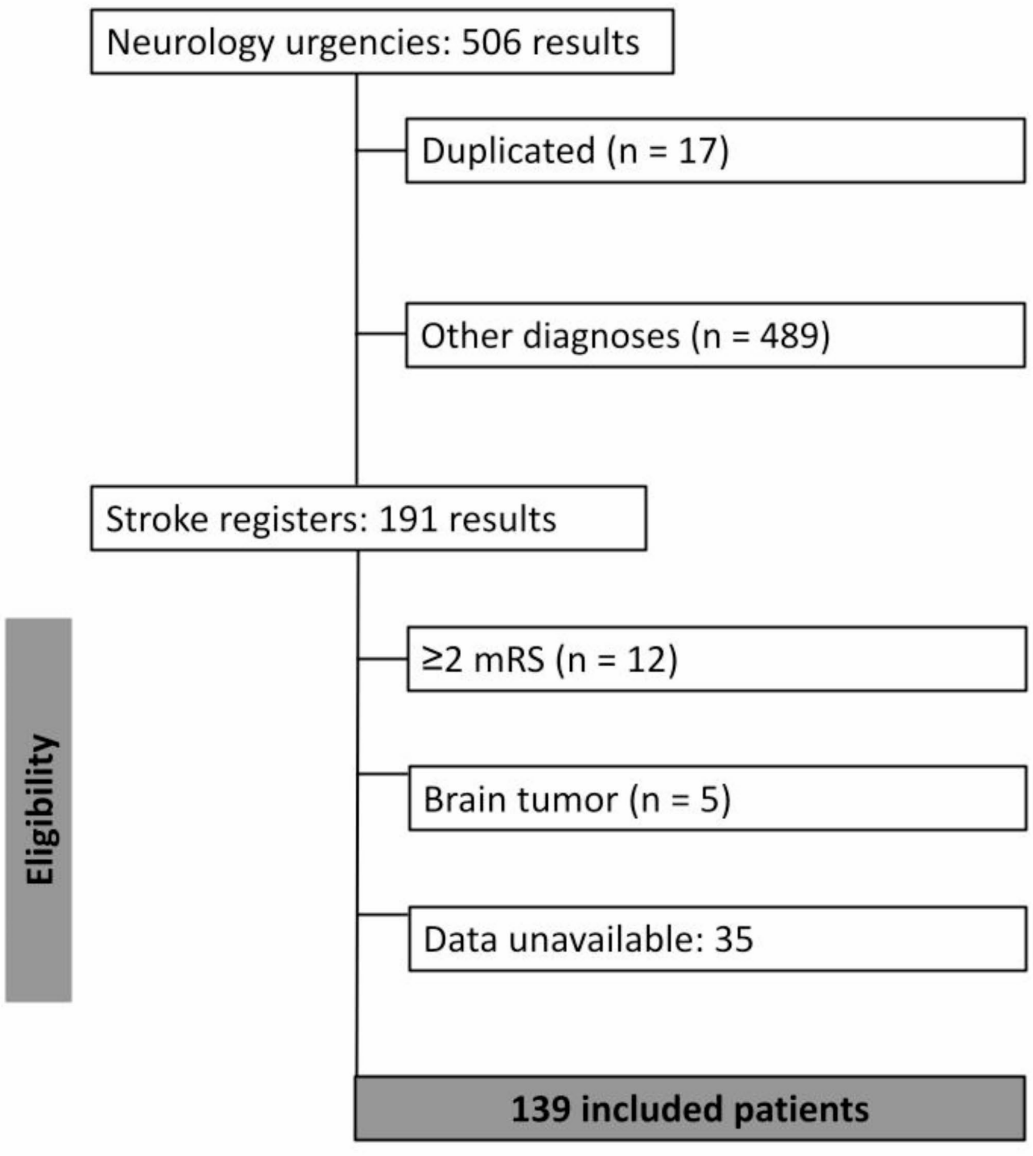
Flow diagram of included patients in the study.

During the study, out of 191 patients observed, 52 were excluded based on previously mentioned study criteria, resulting in a total of 139 participants included in this study (Fig. 1).

Sociodemographic characteristics stratified by stroke subtype are summarized in Table 1. Patients’ mean age was 59.5 ± 13.5 years, with a range from 34 to 101 years. They were divided into age groups. Among the patients, 91 (66%) were male. Male stroke patients were younger than females (57.6 ± 12.5 years vs. 63.1 ± 14.7 years; *p* = 0.045).

**Table 1:** Patients’ characteristics and stroke subtypes.

Characteristics	N 139	Stroke subtype		*p*
		Ischemic 102	Hemorrhagic 37	
Sex				
Male n (%)	91 (65.5)	64 (62.7)	27 (73.0)	0.037*
Age (years)^†^	59.5 ± 13.5	61.6 ± 13.3	53.6 ± 12.3	0.001*
≤ 39	9 (6.5)	4 (3.9)	5 (13.5)	
40–49	26 (18.7)	14 (13.7)	12 (32.4)	
50–59	38 (27.3)	27 (26.5)	11 (29.7)	
60–69	35 (25.2)	31 (30.4)	4 (10.8)	
≥ 70	31 (22.3)	26 (25.5)	5 (13.5)	
Length of stay^†^	8.3 ± 4.7	7.4 ± 3.8	10.8 ± 6.1	0.001*
Onset-to-door time (hours)^‡^	14	24	8	0.035*
≤ 3 h	15 (10.8)	11 (10.8)	4 (10.8)	
Systolic blood pressure^†^	171 ± 32.2	166.4 ± 30.3	183.8 ± 34.2	0.004*
> 139 mmHg n (%)	118 (86.1)	84 (83.2)	34 (94.4)	
Diastolic blood pressure^†^	97.8 ± 19.2	92.3 ± 17.4	113.4 ± 15.4	< 0.001*
≥ 90 mmHg n (%)	89 (65.0)	55 (54.5)	34 (94.4)	
mRS score				0.255
≤ 2	70 (50.4)	53 (52.0)	17 (45.9)	
3–4	61 (43.8)	46 (45.1)	15 (20.5)	
> 4	8 (5.8)	3 (2.9)	5 (13.5)	

*mRS* modified rankin scale.

*Statistical significance data.

^†^Data presented is mean ± standard deviation.

^‡^Data presented is medians.

Most of the patients had ischemic stroke (102 [73.4%]). There were two primary forms of hemorrhagic stroke: 34 (91.9%) were intracerebral hemorrhage (ICH), and 3 (8.1%) subarachnoid hemorrhage (SAH). A comparison of age by stroke subtype, revealed that those with hemorrhagic type had a significantly lower mean age (Table 3).

**Fig. 2:**
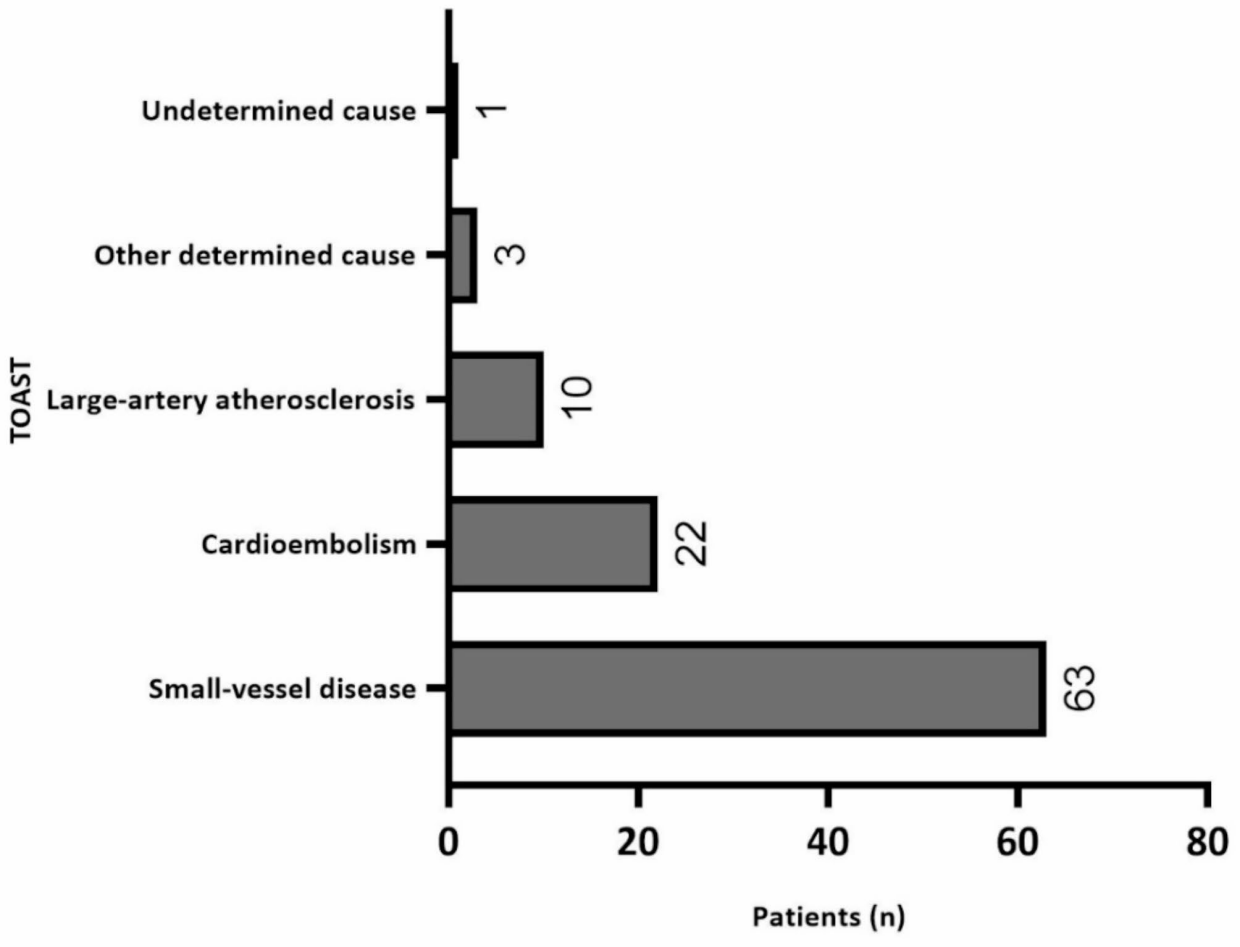
TOAST classification for ischemic stroke. TOAST—Trial of Org 10172 in acute stroke treatment.

Etiological mechanism of ischemic stroke was assessed using the Trial of Org 10,172 in Acute Stroke Treatment (TOAST). Out of 99 patients with ischemic stroke, we observed 63 (63.6%) patients with small-vessel disease (SVD), followed by cardioembolism with 22 (22.2%) and 10 (10.1%) had large-artery atherosclerosis (LAA; Fig. 2).

Prevailing risk factors included hypertension in 130 (96%) and alcohol use in 91 (67.4%). Patients with smoking history (*p* = 0.009) and diabetes (*p* = 0.005) demonstrated a higher likelihood of presenting ischemic stroke compared to hemorrhagic stroke. However, no statistically significant association was found between the remaining risk factors and a particular type of stroke (Table 2). Only four of our research participants did not present any risk factors.

**Table 2:** Risk factors and stroke subtypes.

Risk factor	N 135	Stroke subtype		*p*
		Ischemic 99	Hemorrhagic 36	
Hypertension n (%)	130 (96.3)	97 (98.0)	33 (91.7)	0.118
Alcohol use n (%)	91 (67.4)	67 (67.7)	24 (66.7)	1.000
Diabetes n (%)	40 (29.6)	36 (36.4)	4 (11.1)	0.005*
Smoking n (%)	30 (22.2)	28 (28.3)	2 (5.6)	0.009*
Dyslipidemia n (%)	28 (20.7)	25 (25.3)	3 (8.3)	0.053
Obesity n (%)	18 (13.3)	14 (14.1)	4 (11.1)	0.780
Fibrillation n (%)	10 (7.2)	9 (9.1)	1 (2.8)	0.289
Heart failure n (%)	5 (3.7)	5 (5.1)	–	0.324

*Statistical significance data.

Median time from stroke onset to hospital admission was 14 h, and only 15 (10.8%) patients arrived at the hospital ≤ 3 h since the first symptom. Mean length of stay was 8.3 ± 4.7 days and patients with hemorrhagic stroke had longer hospital stays (*p* = 0.001) (Table 1).

The mRS was used to assess the degree of patients’ disabilities at discharge moment in-person. A majority of patients had a mild disability with a total of 70 (50.4%) (Table 1). We also found a statistically significant association between the mRS score and the occurrence of complications. Patients who experienced complications during their hospital stay had higher mRS scores and therefore more severe disability (*p* < 0.001) (Table 3).

**Table 3:** Length of stay, stroke subtype, complications and modified Rankin scale’s score.

	N (%)	mRS			*p*
		≤ 2	3–4	> 4	
Length of stay^†^	139	8 ± 4	9 ± 4	12 ± 11	0.001*
Stroke subtype n (%)	139	70 (50.4)	61 (43.8)	8 (5.8)	0.255
Ischemic	102 (73.4)	53 (52.0)	46 (45.1)	3 (2.9)	
Hemorrhagic	37 (26.6)	17 (45.9)	15 (40.5)	5 (13.5)	
Complications n (%)	139	70 (50.4)	61 (43.8)	8 (5.8)	< 0.001*
Yes	45 (32.4)	12 (26.7)	27 (60.0)	6 (13.3)	
No	94 (67.6)	58 (61.7)	34 (36.2)	2 (2.1)	

*mRS* modified Rankin scale.

*Statistical significance.

^†^Data presented is mean ± standard deviation.

Middle cerebral artery (MCA) was the most impaired vascular territory in 105 (80.8%) patients, followed by the pontine arteries in 10 (7.7%) patients, vascular (Table 4).

**Table 4:** Vascular territory and stroke subtype.

Vascular territory	N (%) 130	Stroke subtype		*p*
		Ischemic 98	Hemorrhagic 32	
Middle cerebral artery	105 (80.8)	75 (76.5)	30 (93.8)	0.055
Pontine arteries	10 (7.7)	10 (10.2)		
Posterior cerebral artery	6 (4.6)	5 (5.1)	1 (3.1)	
Anterior cerebral artery	5 (3.8)	4 (4.1)	1 (3.1)	
Superior cerebellar artery	3 (2.3)	3 (3.1)		
Posterior cerebellar artery	1 (0.8)	1 (1.0)		

There was a statistically significant difference between admission blood pressure measurements and stroke subtype (*p* < 0.01), the means were consistently higher in hemorrhagic stroke (Table 1).

It is important to emphasize that out of all patients only 45 (32%) experienced clinical complications during their hospital stay. The most frequent complications were obstipation 15 (33%), urinary tract infections (UTIs) 11 (24%), and respiratory infections 8 (18%).

All patients were treated in a general medical ward due to the unavailability of stroke unit facilities in the country.

## Discussion

This was a cross-sectional study of stoke patients in a tertiary Angolan hospital setting. Angola is a lower-middle income country with health issues characterized by infectious diseases such as malaria, tuberculous, HIV/AIDS, car accidents, and maternal and perinatal health challenges. Currently, Angola is experiencing substantial changes with the increase of chronic noncommunicable diseases associated with poor control of risk factors for cardiovascular and cerebrovascular diseases such as hypertension, dyslipidemia, and diabetes. Consequently, the incidence of stroke is increasing with alarming concerns reaching active African young people under the age of 60.

Our study showed that our patients were significantly younger than what has been reported in most of the studies previously published involving working-age people, but similar to some African studies in Ethiopia^11^ and Kinshasa^12^. A recent meta-analysis^13^ showed that LMICs tend to have younger stroke patients than those from high-income countries, with mean ages of 63.1 and 68.6 years respectively. This can be attributed to disparities in the quality of health services, low-to-middle-income countries, for instance, have insufficient resources and their citizens face challenges in accessing health services. All these factors have devastating effects on the prevention and management of stroke.

Although there has been a shift in the worldwide occurrence and frequency of stroke, with a greater number of women being affected, as reported in the last global burden of diseases study^14^, our research revealed that stroke was more prevalent in men and at younger ages. These data are consistent with a previous study^15^ in which 66% of stroke patients were male and contrasts with another study^16^ with a total of 104 patients, where the population was evenly distributed between women and men, with 50% each. We believe that the highest occurrence among males may be due to an unwillingness to seek hospital treatment or follow medical advice while suffering from hypertension or another cardiovascular risk factor, which is frequently seen as cultural practice. In Africa, males are generally family heads, and a stroke disrupts the economic stability of these households.

Patients, mainly those suffering from ischemic stroke, arrived at the hospital with very extended stroke onset times. Only a minority of individuals arrived at the hospital within three hours. These findings are deeply concerning since time is critical for stroke management, initiating therapy and achieving improved outcomes. Most ischemic stroke patients do not receive reperfusion therapy. Previous research^17^ in Seoul found that patients who arrived within 4.5 h of stroke onset had improved clinical outcomes, despite stroke severity, pre-stroke improvement, or even stroke subtype.

Most of the patients had mild disability according to the mRS, but those who had complications during hospitalization had higher scores and worse disability and dependency. A multicenter trial^18^ showed that complications such as infections increased the likelihood of dependency measured by mRS, and a similar pattern was found for the length of stay, patients with higher scores stayed longer in the hospital. A similar study in Burkina Faso^19^, revealed that severity of stroke prolonged the length of hospital stay.

Ischemic stroke was the prevailing subtype, as confirmed by similar studies that reported a prevalence of 76.5%^20^ and 86.5%^16^. Hemorrhagic stroke affected younger patients, which is consistent with results from earlier studies in which hemorrhagic stroke was more common in middle-aged people and decreased in the elderly^20–22^. Hypertension, sedentarism, and other common stroke risk factors, particularly hypertension, which is presently considered to be the leading risk factor for hemorrhagic stroke, are on the rise among young adults^23–25^.

Ischemic stroke TOAST classification revealed that the most frequent etiology was SVD, which differs from studies in Brazil^26^ and Indonesia^27^ that found LAA as more common. The SIREN study^28^ conducted in Nigeria and Ghana revealed that in patients younger than 50 years LAA was more common, while in those 50 and older SVD was more prevalent. SVD incidence is increasing in black patients according to some studies^29,30^ and can be caused by hypertension. Almost all patients in the present study were hypertensive (96%) and higher blood pressure was associated with hemorrhagic stroke. These findings are similar with a study in which 86% of the participants were hypertensive^31^. In a study^32^ conducted in Angola on hypertension screening, 4844 individuals (33.6%) were hypertensive. Among them, only half were aware of their diagnosis, and of those, only 46.3% were taking antihypertensive medication. Additionally, only 42.6% of all hypertensive individuals had hypertension under control. These worrying data point out the persistent challenges in this country regarding the management and diagnosis of hypertension, a critical preventive measure against stroke. In Africa, the annual stroke incidence can reach as high as 316 per 100,000 people, with hypertension remaining the most important modifiable risk factor^33^.

We also found an association between ischemic stroke and other risk factors (diabetes and smoking). It was impossible to ignore the prevalence of the remaining risk factors, which play an important role in the occurrence of stroke. All of this leads us to believe that the problem results from a failure of primary prevention, resulting in young patients with multiple risk factors and an increased risk of having severe stroke. This has a negative impact on both the patients and the country’s socioeconomic burden, particularly since these individuals are economically active, which creates a cycle that must be stopped. Given the magnitude of the problem, as demonstrated by previous studies^9,34^ on the financial impact of stroke, every effort must be made to prevent it.

Despite a wide range of clinical manifestations, the primary symptoms observed were motor deficits and speech impairment. This finding corresponded with the results of a related study^35^, which reported that weakness in the lower and upper limbs were the most common symptoms, followed by dysarthria and aphasia. Some participants in our research experienced important sequelae, as well as concerns about returning to work.

## Conclusion

The study revealed that Angolan stroke patients were younger than previously reported in the literature. Men were the most affected, highlighting a substantial economic impact on households. Ischemic stroke was the most prevalent subtype, with SVD as the main etiology. Hemorrhagic stroke had the most significant impact on younger patients. MCA was the most affected vascular territory in both types of stroke.

Patients arrived at hospital with prolonged stroke onset-time and had mild to moderate disability. Those who experienced clinical complications had increased grades of disability and dependency. Hypertension was the most common risk factor. A minority of patients experienced clinical complications during their hospitalization; those who did were primarily afflicted with obstipation and urinary and respiratory tract infections. It is essential to enhance stroke prevention measures due to the increasing occurrence of strokes at younger ages throughout time and the rising frequency of risk factors.

### Limitations

As we conducted a retrospective analysis, our research was limited by the data that was available resulting in lack of topographic features, race data, some risk factors of stroke and may be some risk of bias. This study took place in a single center in Angola; therefore, it may not be entirely representative of the country’s other hospitals.

This hospital-based study also has a selection bias, where the hospital tends to predominantly receive the most severe cases.

## Data Availability

The data related to this study are available from the corresponding author upon reasonable request.
